# TSWIFT, a novel method for iterative staining of embedded and mounted human brain sections

**DOI:** 10.1038/s41598-024-63152-2

**Published:** 2024-06-03

**Authors:** Corey M. Porter, Sarah Tabler, Sooin Choi, Matthias C. Truttmann

**Affiliations:** 1 Department of Molecular and Integrative Physiology, University of Michigan, BSRB, 109 Zina Pitcher Place, Ann Arbor, MI 48109 USA; 2 Geriatrics Center, University of Michigan, Ann Arbor, MI 48109 USA

**Keywords:** Biological techniques, Neuroscience

## Abstract

Comprehensive characterization of protein networks in mounted brain tissue represents a major challenge in brain and neurodegenerative disease research. In this study, we develop a simple staining method, called TSWIFT, to iteratively stain pre-mounted formalin fixed, paraffin embedded (FFPE) brain sections, thus enabling high-dimensional sample phenotyping. We show that TSWIFT conserves tissue architecture and allows for relabeling a single mounted FFPE sample more than 10 times, even after prolonged storage at 4 °C. Our results establish TSWIFT as an efficient method to obtain integrated high-dimensional knowledge of cellular proteomes by analyzing mounted FFPE human brain tissue.

## Introduction

Mammalian tissue is comprised of a diverse mixture of cell types, each expressing its own characteristic set of proteins. Immunohistochemistry and immunofluorescence imaging are among the most common and appropriate tools to study this complex diversity in human tissue. However, these techniques are limited in scope by the number of proteins that can be probed (stained) at one time. The introduction of methods relying on antibody-dye conjugates^1^, antibody-oligonucleotide conjugates^2–4^, mild tissue stripping, optimized fluorophore usage, and/or horseradish peroxidase (HRP)-reactive fluorophores have increased the number of proteins that can be stained and visualized simultaneously. However, the reliance on unconventional antibody conjugates, equipment, and proprietary dyes prevents a broader application of these methods. The recent development of elaborate tissue delipidation and preservation techniques offers a convenient solution to most of these restrictions. One of these methods is SHIELD (**S**tabilization under **H**arsh conditions via **I**ntramolecular **E**poxide **L**inkages to prevent **D**egradation), a technique established to preserve free-floating tissue sections and tissue pieces^5^. This process involves first creating strong intra- and intermolecular bonds with a flexible polyepoxide, which strengthens tissue architecture without interfering with protein epitopes or changing tissue morphology, allowing for repeated cycles of staining. Then, the tissue is delipidated, reducing background fluorescence. Following SHIELD treatment, a single tissue sample can be iteratively stained, imaged, destained, and re-stained using conventional antigen-specific primary antibodies and dye-conjugated secondary antibodies^5^. SHIELD and related concepts have been successfully used to prepare large tissue samples (e.g., whole mouse brains), fixed and fixed-frozen cells in multi-well plates^1,6,7^, and non-mounted frozen tissue sections. However, many archival samples provided by human tissue banks are formalin fixed, paraffin embedded (FFPE), and are often pre-cut and mounted onto glass slides. This makes these samples incompatible with currently available tissue delipidation and iterative staining methods.

In this study, we describe TSWIFT (**T**issue Staining **W**ith **I**terative **F**luorescent **T**agging), a novel tissue processing and staining method to iteratively stain mounted FFPE brain tissue on glass slides. TSWIFT preserves protein antigens in mounted tissues and integrates the signal amplification aspect of using primary and secondary antibodies. We show that TSWIFT permits more than 10 subsequent staining-destaining-restaining cycles of a single human brain section, even when stored up to 6 months between consecutive processing cycles. Taken as a whole, we establish TSWIFT as the first tissue processing and staining method optimized for mounted FFPE human brain tissue. This method allows us to obtain high-dimensional representations of cellular proteomes in human brain sections at subcellular resolution, which may accelerate our understanding of pathophysiological processes in the human brain.

## Methods

### Tissue sections

FFPE tissue samples were provided by the University of Michigan Brain Bank. Tissues were fixed in 10% neutral buffered formalin for a minimum of 14 days, before they are sectioned at brain conference, processed into paraffin blocks, and stored at room temperature. Samples were cut into 5 µm thick sections onto Fisherbrand Tissue Path Superfrost Plus Gold slides and baked at 65 °C for 1 h. Sample details are summarized in supplementary Table S1.

### SHIELD FFPE preparation

The SHIELD FFPE procedure was adapted from Park et al.^5^. Mounted FFPE brain sections were heated at 60 °C for 10 min before being deparaffinized in xylene, hydrated in 100%, 95%, then 70% ethanol followed by deionized water. Slides were then incubated in a 7:1 mix of SHIELD-ON buffer and SHIELD Epoxy solution from LifeCanvas Technologies Inc. Slides were incubated in this solution for 6 h at 4 °C with gentle shaking, then moved to room temperature for 24 h with continued shaking. Tissue delipidation was performed in SDS clearing solution (300 mM sodium dodecyl sulfate, 10 mM boric acid, 100 mM sodium sulfate, brought to pH 9.0 with sodium hydroxide) at 37 °C with shaking for 24 h. Slides were then incubated in phosphate or tris-buffered saline with 0.2% Triton X-100 (PBSX/TBSX) overnight at 37 °C to remove SDS.

### Iterative Immunostaining

Antigen retrieval was done in citrate buffer (10 mM citric Acid, 0.05% Tween 20, pH 6.0) in a Rosewill steamer model RHST 15001 for 25 min. Slides were then washed in Tris Buffered Saline (TBS) once. Sections were blocked in 10% FBS, 10%BSA with 0.2% Triton X-100 in TBS for 1 h at room temperature with shaking. Primary antibodies (see supplementary Table S2) were diluted in 5% BSA, 0.2% Triton X-100 in TBS, covered with a small piece of parafilm, and incubated overnight at 4 °C. Slides were washed 3x in TBS before applying secondary antibodies. Secondary antibodies were diluted in the same buffer as primary antibodies, along with DAPI (4′,6-diamidino-2-phenylindole) at 50 ug/ml and incubated on sections in the same manner as the primary antibodies for 1 h at room temperature. To prevent autofluorescence from any lipofuscin remaining post lipid-clearing, 0.5% Sudan Black in 70% ethanol was applied for 10 min. Coverslips were applied to tissue sections using ProLong Diamond Antifade Mountant (Invitrogen) and allowed to cure overnight before imaging. After imaging, coverslips were removed by incubating slides in 70% ethanol at room temperature or TBS at 37 °C before destaining in destaining buffer as described in Park et al.^5^ (7 mM SDS, 0.5M Tris HCl, pH6.8 with 1% 2-mercaptoethanol add fresh before destaining) at 37 °C for 18 h. Slides were washed three times 30 min in TBS before beginning the next round of staining, starting with the blocking step. Different combinations of primary and secondary antibodies were used throughout this study to demonstrate that the dye molecule attached to the secondary antibody does not affect the staining and destaining process.

### Image acquisition and analysis

Stained slides were imaged using a Keyence BZ-X700 Microscope equipped with a CFI Nikon plain Apo 20x objective. Exposure times for each channel were set using the auto-exposure function in the BZ-X software and kept constant for the entire imaging cycle. Image analysis was done using BZ-X Analyzer and BZ-X Wide image Viewer from Keyence. Cyclic stains were analyzed by comparing small areas of the tissue in subsequent stains to first determine the viability of our approach. Once the technique was validated, small areas of each cycle of staining on the same tissue were compared to determine which markers co-localize. To facilitate visual inspection of imaging results, color levels were automatically enhanced using the BZ-X Analyzer software, if necessary. Image intensity profiles were extracted using the “Plot Profile” feature in Fiji^8^ and normalized (mean subtraction) before plotting.

### Data availability and sharing

All original high-quality image files presented in this study will be deposited on the Deep Blue Data repository (https://deepblue.lib.umich.edu/data) and made available for download upon registration.

## Results

### TSWIFT allows for repeated staining and destaining with signal pattern retention

**Figure 1:**
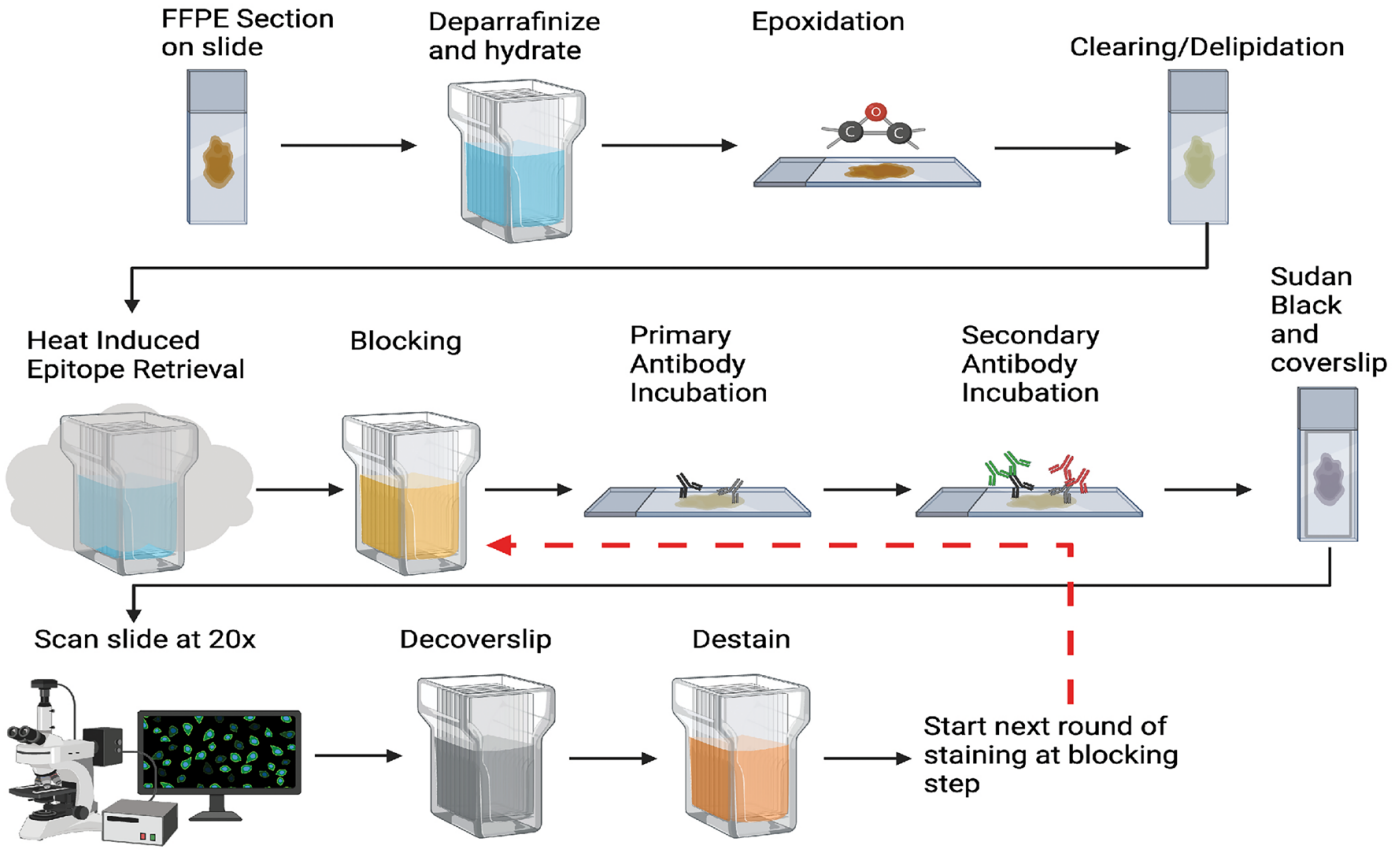
Schematic overview. A schematic representation of the TSWIFT protocol. Deparaffinization and rehydration are followed by epoxidation using SHIELD ON and Epoxy buffers to strengthen the tissue. Sections are then delipidated to reduce autofluorescence, followed by washing and a traditional immunofluorescence protocol including Sudan Black. The entire section is scanned at 20 × before decoverslipping, destaining, and restarting the staining process at the blocking step.

To establish an easily adaptable technique for iterative staining of mounted FFPE sections, we developed a new tissue clearance and staining protocol, incorporating commercially available SHIELD reagents (Fig. 1)^5^. We first deparaffinized and rehydrated 5 µm FFPE brain sections before epoxidation using SHIELD ON and SHIELD Epoxy buffers to strengthen tissue sections to withstand consecutive staining cycles. Next, we delipidated sections for 24 h to reduce autofluorescence. Following a washing cycle, we then followed a traditional immunofluorescence staining protocol using Sudan Black (to reduce background fluorescence; see supp. figure 1), unconjugated primary antibodies, and fluorophore conjugated secondary antibodies.

**Figure 2:**
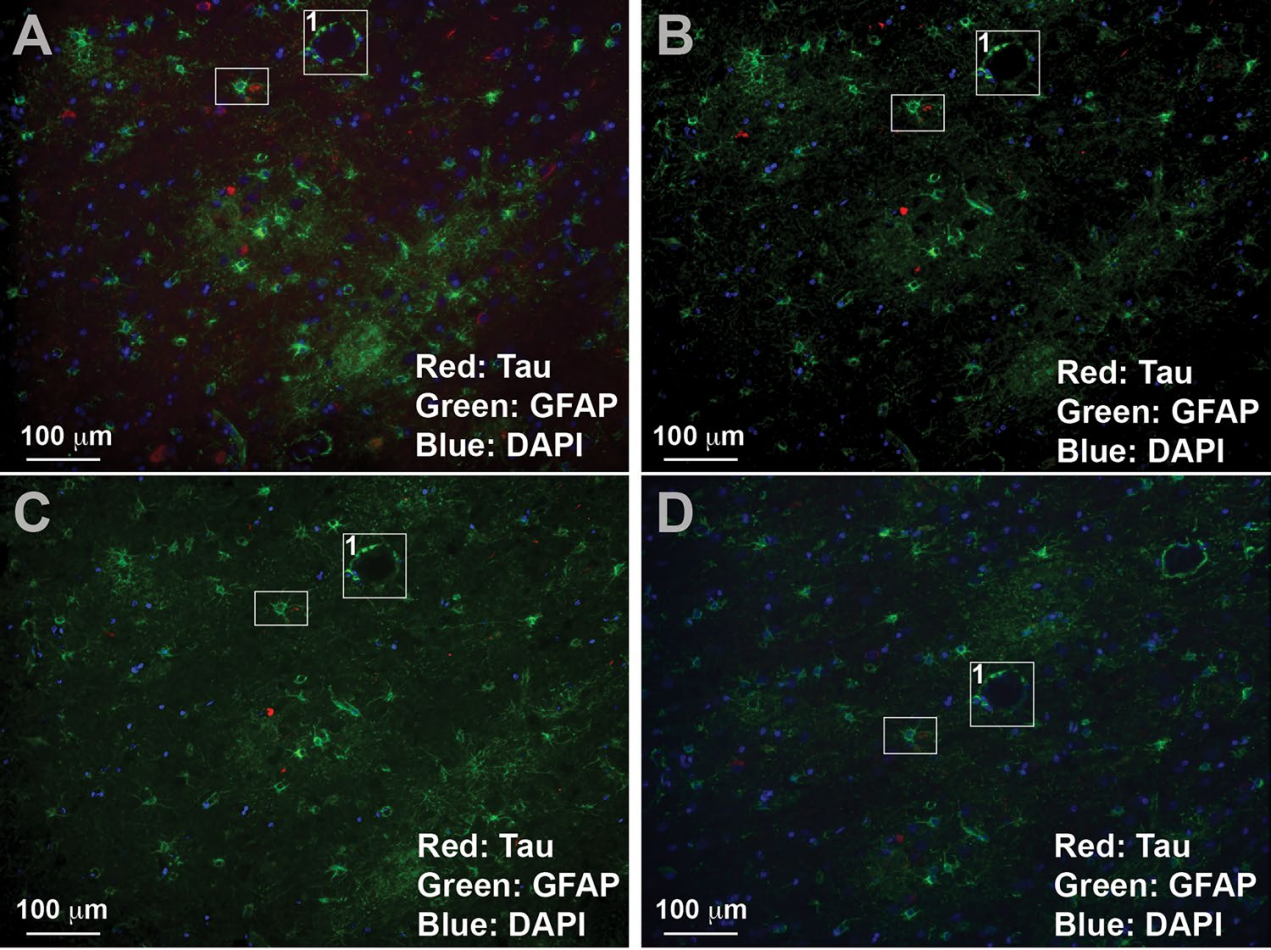
SHIELD protects tissue and allows for repeated staining of tissue with maintained signal. 20 × images from cycles of staining post-SHIELD. All are stained with DAPI at 50 µg/mL (Blue), Santa Cruz sc-58860 mouse mAb Tau (secondary 568) (Red) and DAKO Z0334 rabbit pAb GFAP (secondary 488) (Green). (**A**): cycle 1, (**B**): cycle 4, (**C**): cycle 7, (**D**): cycle 9.

**Figure 3:**
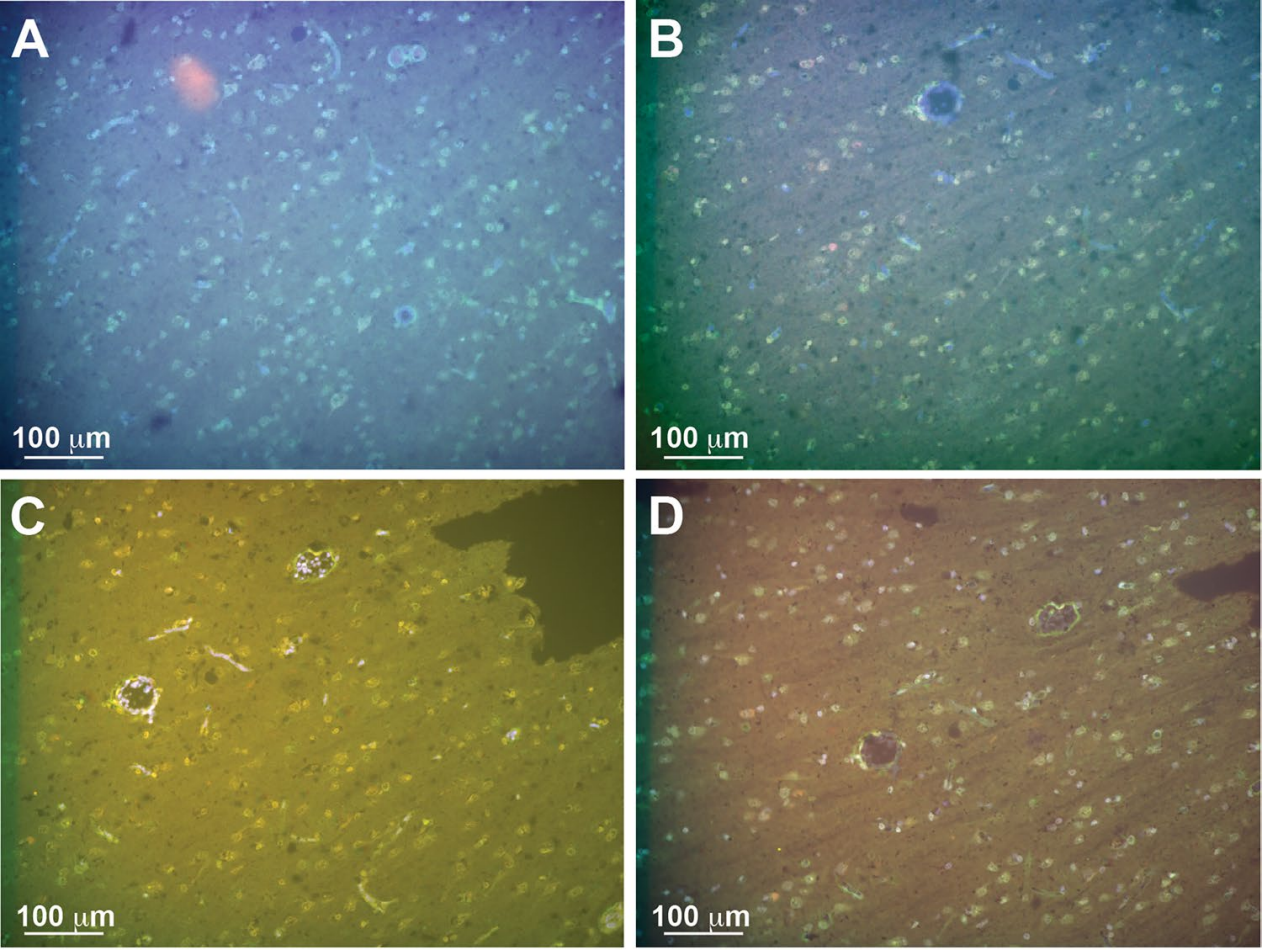
Destained sections show little to no retention of staining. Overlay image of individual channels taken at very long exposure times (up to 3.5 s) confirm efficient tissue destaining and minimal tissue degradation. 20 × images from destain cycles of staining post-SHIELD; (**A**): cycle 1 destain, (**B**): cycle 4 destain, (**C**): cycle7 destain, (**D**): cycle 9 destain.

**Figure 4:**
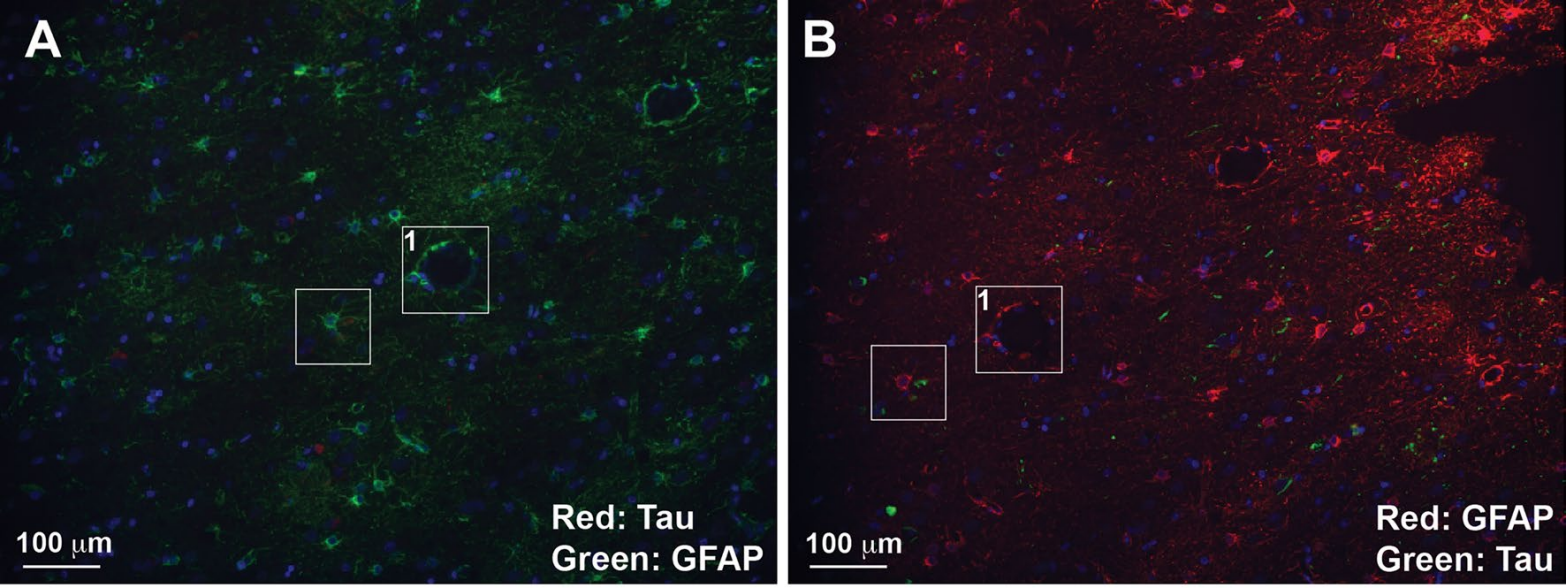
Residual secondary antibody deposits are not contributing to signal pattern. Images from cycle 9 and 10 of proof-of-concept staining. All are stained with DAPI, Santa Cruz sc-58860 mouse mAb Tau and DAKO Z0334 rabbit pAb GFAP. (**A**): cycle 9 has Tau in red (568) and GFAP in green (488) . (**B**): cycle 10 has Tau in green (488) and GFAP in red (568).

**Figure 5:**
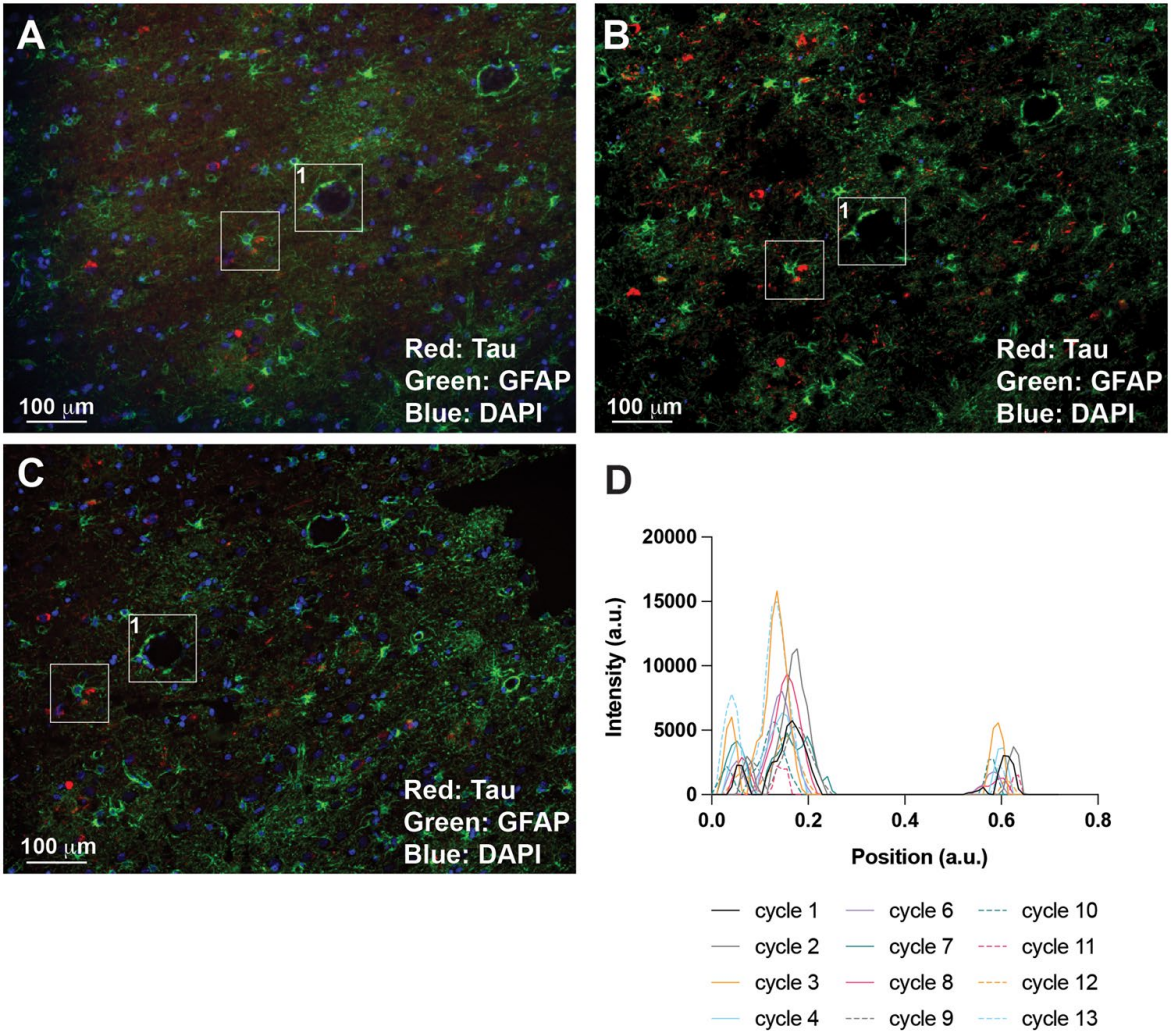
Sample storage at 4 °C does not impair processing. 1 month storage at 4 °C does not decrease staining, while 4-month storage decreases signal but maintains staining pattern. All are stained with DAPI (Blue), Santa Cruz sc-58860 mouse mAb Tau (secondary 568) (Red), and DAKO Z0334 rabbit pAb GFAP (secondary 488) (Green). (**A**): cycle 9 (before 4 °C storage) (**B**): cycle 11 (after 1 month 4 °C storage) (**C**): cycle 13 D: cycle 14 after 4 months at 4 °C. (**D**): Intensity profile of DAPI signal in box 1 across cycles.

To validate our new TSWIFT protocol on mounted FFPE sections, we conducted a series of experiments in which we processed and iteratively imaged, destained, imaged, and restained an AD high (Braak VI) cortex FFPE section, with the same primary and secondary antibodies (DAKO anti-GFAP; secondary fluorophore 488, Santa Cruz anti-Tau; secondary fluorophore 568) (Fig. 2, supp. figure 2–7). We found that even after 10 staining-destaining cycles, epitope patterns were well retained (Fig. 5D, supp. figures 2–7). Control imaging after each destaining step confirmed that little to no signal was retained (Fig. 3). Staining with only secondary antibodies in the fifth cycle and again using primary antibodies paired with secondary antibodies coupled to Alexa488 in the tenth staining cycle further confirmed efficient removal of antibodies during the destaining process (supp figures 4 and 6, Fig. 4). After destaining after cycle 10, we stored the test slide for 1 month at 4 °C in TBS supplemented with 0.02% sodium azide. After this storage period, we stained and imaged the sample and found that epitope signal patterns were still intact (Fig. 5A–D). Because of repeated coverslip removals, we observed minor sample disruptions (i.e., tears in the tissue section). Testing several cover slip removal methods, we found that removing the coverslip by soaking the slide in a PBS bath at 37 °C with gentle shaking resulted in minimal tissue integrity loss. Together, these results establish that TSWIFT is an effective approach to iteratively stain mounted FFPE tissue sections with minimal signal loss and tissue disruption.

### Iterative staining allows for co-localization analysis of multiple targets

**Figure 6:**
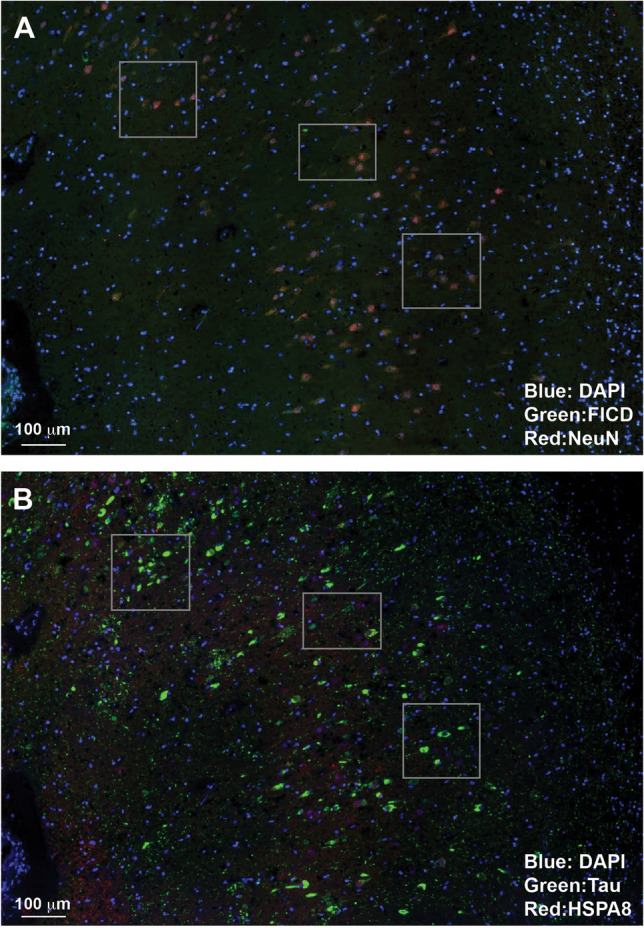
Tau only partially co-localizes with neuronal markers. Comparing cycles of staining shows that NeuN (neuronal nuclear marker) and Tau multiple isoforms (present in axons of neurons, astrocytes, and oligodendrocytes) co-localize to a small degree, with Tau being more evenly distributed in an area of a hippocampus near the dentate gyrus from a patient with Braak V/VI AD than NeuN. FICD is sparsely expressed in this area, and HSPA8 is not expressed in this area of the hippocampus. A section of hippocampus from an individual with Alzheimer’s Disease Braak V/VI was iteratively stained using the SHIELD protocol. Subsequent cycles are shown, with boxes highlighting corresponding areas. All cycles were stained with DAPI (Blue). (**A**): Cycle 1 Santa Cruz sc-515368 mouse mAb FICD clone G-7 (secondary 488) (Green) and ProteinTech 26,975–1-AP rabbit pAb NeuN (secondary 568) (Red) (**B**): Cycle 2 DAKO A0024 Rabbit pAb Pan human tau (multiple isoforms) (secondary 488) (Green) and Santa Cruz 7298 mouse mAb HSPA8 (secondary 568) (Red).

**Figure 7:**
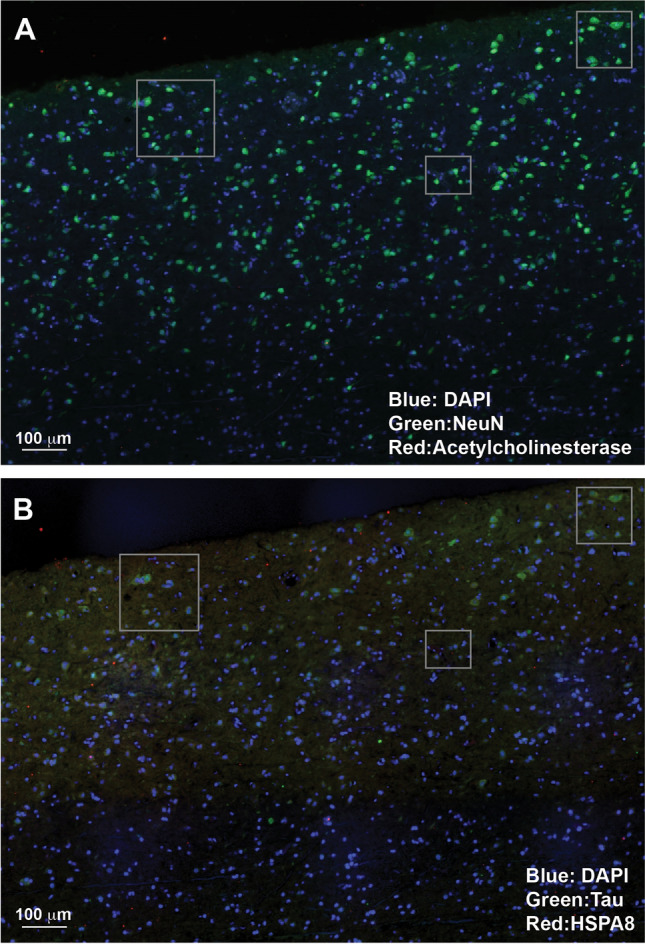
Tau only partially co-localizes with neuronal markers. Comparing cycles of staining shows that NeuN (neuronal nuclear marker) and Tau multiple isoforms (present in axons of neurons, astrocytes, and oligodendrocytes) co-localize to a moderate degree in an area of cortex from an individual with no neurological diagnosis. NeuN is more widely expressed in this region, but is more concentrated in the grey matter, as is Tau. Acetylcholinesterase (ACHE, cholinergic neuron marker) and HSPA8 are less abundant but are more concentrated in the grey matter as well. A section of cortex from an individual with no neurological diagnosis was iteratively stained using the SHIELD protocol. Subsequent cycles are shown, with boxes highlighting corresponding areas. All cycles were stained with DAPI (Blue). (**A**): Cycle 2 ProteinTech 26975-1-AP rabbit pAb NeuN (secondary 488) (Green) and Developmental Studies Hybridoma Bank tor23 mouse anti-Acetylcholinesterase, presynaptic (secondary 568) (Red). (**B**): Cycle 4 DAKO A0024 Rabbit pAb Pan human tau (multiple isoforms) (secondary 488) (Green) and Santa Cruz 7298 mouse mAb HSPA8 (secondary 568) (Red).

The main advantage of iterative staining is that it allows for the detection of more targets on the same piece of tissue compared to standard immunohistochemistry or immunofluorescence. These multiple images can then be used to determine how the various targets correspond to one another in the same sample. We compared images from subsequent staining cycles from an AD Braak IV/V hippocampus stained with anti-Neu antibody (secondary fluorophore 568) and anti-FICD antibody (secondary fluorophore 488) in round 1 and anti-Tau (multiple isoforms) antibody (secondary antibody 488) and anti-HSPA8 antibody (secondary fluorophore 568) used in cycle 2. In these images, we observed limited colocalization of NeuN and Tau (Fig. 6). This may be because Tau is normally expressed in the soma, whereas NeuN is located near the nucleus^9,10^. Additionally, in dying neurons, where Tau is more likely to be detected in the soma, NeuN expression is diminished^9,10^. FICD is sparsely expressed in this area, and HSPA8 is not expressed in this area of the hippocampus (Fig. 6). We also compared cycles of staining from an area of cortex from an individual with no neurological diagnosis. In cycle 1 the cortex was stained with anti- NeuN antibody (secondary fluorophore 488) and anti-Acetylcholinesterase, presynaptic antibody (secondary fluorophore 568), and with anti- Pan human tau (multiple isoforms) (secondary fluorphore 488) antibody and anti-HSPA8 (secondary fluorphore 568) antibody in cycle 4 (Fig. 7). We found that NeuN and Tau co-localize moderately, but NeuN is expressed more broadly. Both are more concentrated in the grey matter.

## Discussion

In this study, we present a novel method, termed TSWIFT, to iteratively stain and image mounted FFPE-embedded tissue sections of mammalian tissue. Several approaches for multiplexed immunofluorescence imaging have previously been described (reviewed here^11^). Most of these approaches require equipment or consumables that are not necessarily available to all laboratories. Alternative methods for antibody stripping using heat^12^ or detergents^13^, while effective, often lead to FFPE sample detachment from the glass slide. TSWIFT allows for the staining of more than four target proteins in a single section, which is an often-found limit for traditional immunostaining methods using commercial primary and secondary antibodies. Next, TSWIFT can be performed using standard laboratory equipment. The stabilization of samples with epoxy, a core element of the TSWIFT method, prevents tissue detachment even after more than 10 cycles of staining/destaining. Further, the ability to store samples for weeks between staining cycles offers the benefit to revisit previously imaged tissue samples and assess additional targets as they are identified during a study or as part of a revision process. We thus believe that our method improves on previously established approaches for iterative immunofluorescence staining and will find broad application in distinct tissue-based research settings. Since we find that absolute signal intensities for repeated stainings of a single epitope may vary (Fig. 5D), we caution against using intensity measurements between cycles as a determinant for epitope abundance and retention.

As expected, epoxidation strengthens the tissue, making repeat staining with minimal loss of tissue integrity possible. Gentle release of the coverslip further minimizes tissue disruption. However, the conservation of tissue integrity remains a key concern which warrants inspection prior to each staining cycle, as minor tissue tearing is likely to occur. Several software solutions for the location integration between staining cycles have already been developed by other groups, which maximizes the amount of information that can be gained from iterative staining approaches^1,2,14,15^. Most of these software solutions are specifically tailored to the staining technique used. We find that Correlia, developed by Rohde et al.^14^, was the most adaptable to our technique. Based on our experience, the best results with localization integration are achieved if the input images were acquired on a confocal microscope, which may represent an additional constrain to some laboratories. Despite some limitations, this technique has great potential.

### Supplementary Information

Supplementary Legends.

Supplementary Figure 1.

Supplementary Figure 2.

Supplementary Figure 3.

Supplementary Figure 4.

Supplementary Figure 5.

Supplementary Figure 6.

Supplementary Figure 7.

Supplementary Table S1.

Supplementary Table S2.

## Data Availability

The unprocessed raw datasets (images) generated and analyzed during the current study are available from the corresponding author upon reasonable request.

## References

[1] Andrea J (2022). IBEX: An iterative immunolabeling and chemical bleaching method for high-content imaging of diverse tissues. Nat. Protoc..

[2] Sarah B (2021). CODEX multiplexed tissue imaging with DNA-conjugated antibodies. Nat. Protoc..

[3] Jocelyn A (2021). Oligonucleotide conjugated antibody strategies for cyclic immunostaining. Sci. Rep..

[4] Jonathan S (2021). Site-specific antibody fragment conjugates for reversible staining in fluorescence microscopy. Chembiochem Eur. J. Chem. Biol..

[5] Park Y-G (2018). Protection of tissue physicochemical properties using polyfunctional crosslinkers. Nat. Biotechnol..

[6] John Darby C (2022). Characterization of the neurogenic niche in the aging dentate gyrus using iterative immunofluorescence imaging. elife.

[7] Gut G, Herrmann MD, Pelkmans L (2018). Multiplexed protein maps link subcellular organization to cellular states. Science.

[8] Johannes S (2012). Fiji: An open-source platform for biological-image analysis. Nat. Methods.

[9] Tim S, Eckhard M (2014). Transport and diffusion of Tau protein in neurons. Cell. Mol. Life Sci. CMLS.

[10] Gusel’nikova VV, Korzhevskiy DE (2015). NeuN as a neuronal nuclear antigen and neuron differentiation marker. Acta Nat..

[11] Wei Chang Colin T (2020). Overview of multiplex immunohistochemistry/immunofluorescence techniques in the era of cancer immunotherapy. Cancer Commun. (Lond. Engl.).

[12] Daniel P (2009). Antibody elution method for multiple immunohistochemistry on primary antibodies raised in the same species and of the same subtype. J. Histochem. Cytochem. Off. J. Histochem. Soc..

[13] Alexander J (2020). A manual multiplex immunofluorescence method for investigating neurodegenerative diseases. J. Neurosci. Methods.

[14] Rohde F, Braumann UD, Schmidt M (2020). Correlia: An ImageJ plug-in to co-register and visualise multimodal correlative micrographs. J. Microsc..

[15] Dragan M (2021). Whole-brain tissue mapping toolkit using large-scale highly multiplexed immunofluorescence imaging and deep neural networks. Nat. Commun..

