# Supplementary material for: TSWIFT, a novel method for iterative staining of embedded and mounted human brain sections
Source: Sci Rep. 2024 Jun 3;14:12688. doi: 10.1038/s41598-024-63152-2 (PMC11148033; doi:10.1038/s41598-024-63152-2)
Supplement: Supplementary file 1 — Supplementary Legends. [file 41598_2024_63152_MOESM1_ESM.docx]

**Supplementary Figure Captions**

**Supplementary Figure S1. Sudan Black is necessary to reduce background fluorescence.** A: Whole slide scan of cycle 2 of staining on a section of cortex from an individual with no neurological diagnosis stained with DAPI (Blue), ProteinTech 26975-1-AP rabbit pAb NeuN (secondary 488) (Green) and Developmental Studies Hybridoma Bank tor23 mouse mAb Acetylcholinesterase, presynaptic (secondary 568) (Red) using Sudan Black. B: The same section on cycle 3 stained with no primaries or secondaries with no Sudan Black.

**Supplementary Figure S2. SHIELD protects tissue and allows for repeated staining of tissue with maintained signal.** 20x images from cycles of staining and destaining post-SHIELD. All are stained with DAPI (Blue), DAKO Z0334 rabbit pAb GFAP (secondary 488) (Green), and Santa Cruz sc-58860 mouse mAb Tau (secondary 568) (Red) antibodies. Unless otherwise noted, images were taken at the exposure automatically selected by the BZ-X700. Because of this, images of destains were taken at much higher exposure than those of stains to detect low amounts of signal. Fig S2: cycles 1 and 2 stained and destained.

**Supplementary Figure S3. SHIELD protects tissue and allows for repeated staining of tissue with maintained signal.** 20x images from cycles of staining and destaining post-SHIELD. All are stained with DAPI (Blue), DAKO Z0334 rabbit pAb GFAP (secondary 488) (Green), and Santa Cruz sc-58860 mouse mAb Tau (secondary 568) (Red) antibodies. Unless otherwise noted, images were taken at the exposure automatically selected by the BZ-X700. Because of this, images of destains were taken at much higher exposure than those of stains to detect low amounts of signal. Fig S3: cycles 3 and 4 stained and destained.

**Supplementary Figure S4. SHIELD protects tissue and allows for repeated staining of tissue with maintained signal.** 20x images from cycles of staining and destaining post-SHIELD. All are stained with DAPI (Blue), DAKO Z0334 rabbit pAb GFAP (secondary 488) (Green), and Santa Cruz sc-58860 mouse mAb Tau (secondary 568) (Red) antibodies. Unless otherwise noted, images were taken at the exposure automatically selected by the BZ-X700. Because of this, images of destains were taken at much higher exposure than those of stains to detect low amounts of signal. Fig S4: cycles 5 and 6 (5 is secondaries only and was taken at the exposure settings used for cycle 4, images were not taken after the cycle 5 destain).

**Supplementary Figure S5. SHIELD protects tissue and allows for repeated staining of tissue with maintained signal.** 20x images from cycles of staining and destaining post-SHIELD. All are stained with DAPI (Blue), DAKO Z0334 rabbit pAb GFAP (secondary 488) (Green), and Santa Cruz sc-58860 mouse mAb Tau (secondary 568) (Red) antibodies. Unless otherwise noted, images were taken at the exposure automatically selected by the BZ-X700. Because of this, images of destains were taken at much higher exposure than those of stains to detect low amounts of signal. Fig S5: cycles 7 and 8.

**Supplementary Figure S6. SHIELD protects tissue and allows for repeated staining of tissue with maintained signal.** 20x images from cycles of staining and destaining post-SHIELD. All are stained with DAPI (Blue), DAKO Z0334 rabbit pAb GFAP (secondary 488) (Green), and Santa Cruz sc-58860 mouse mAb Tau (secondary 568) (Red) antibodies. Unless otherwise noted, images were taken at the exposure automatically selected by the BZ-X700. Because of this, images of destains were taken at much higher exposure than those of stains to detect low amounts of signal. Fig S6: cycles 9 and 10 ( in round 10, secondary antibody fluorophores were swapped, images were not taken after the cycle 10 destain).

**Supplementary Figure S7. SHIELD protects tissue and allows for repeated staining of tissue with maintained signal.** 20x images from cycles of staining and destaining post-SHIELD. All are stained with DAPI (Blue), DAKO Z0334 rabbit pAb GFAP (secondary 488) (Green), and Santa Cruz sc-58860 mouse mAb Tau (secondary 568) (Red) antibodies. Unless otherwise noted, images were taken at the exposure automatically selected by the BZ-X700. Because of this, images of destains were taken at much higher exposure than those of stains to detect low amounts of signal. Fig S7: cycles 11, 12 and 13 ( no destain images were taken for cycle 12 and 13).
