# Supplementary figures and images for: TSWIFT, a novel method for iterative staining of embedded and mounted human brain sections
Source: Sci Rep. 2024 Jun 3;14:12688. doi: 10.1038/s41598-024-63152-2 (PMC11148033; doi:10.1038/s41598-024-63152-2)

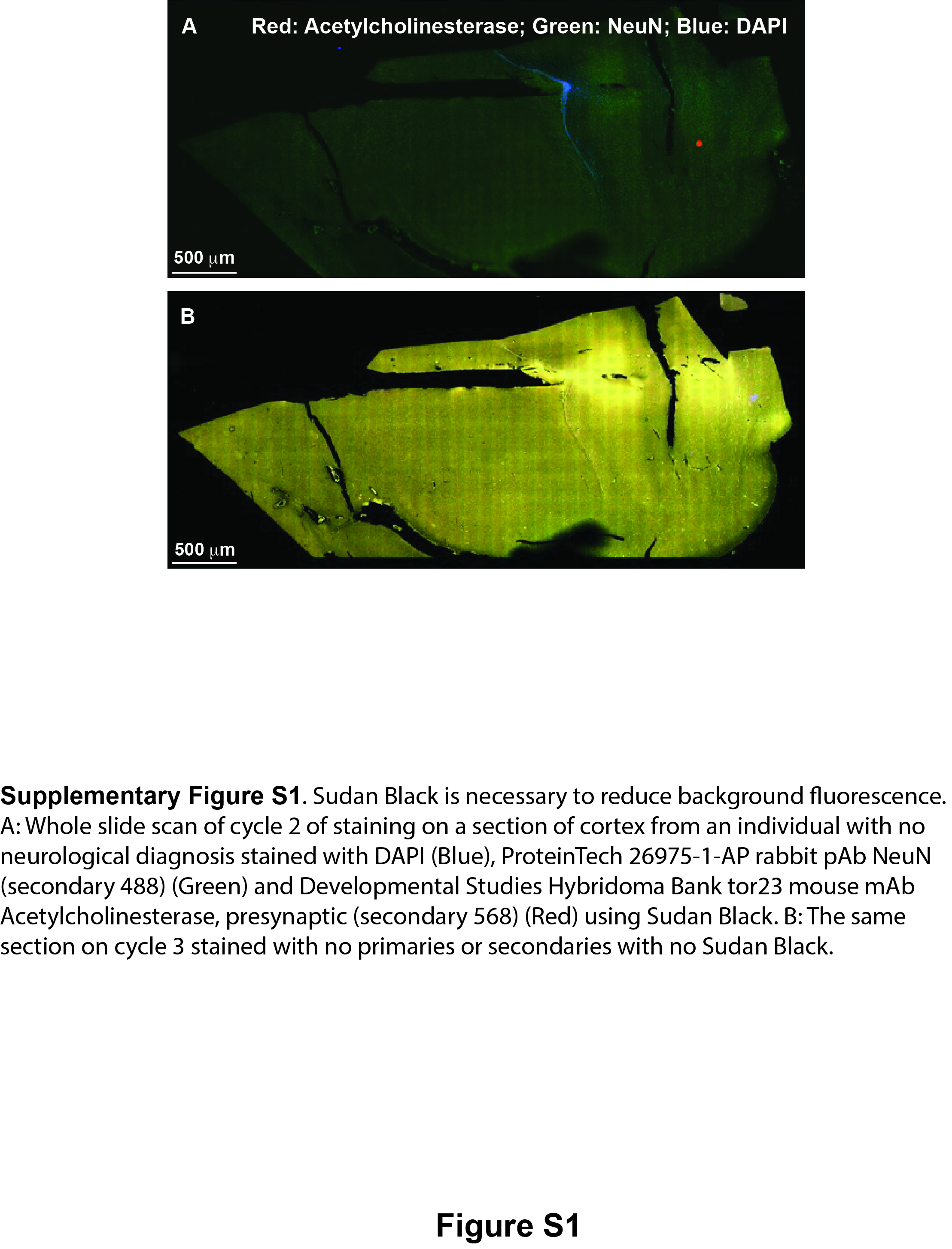

Supplement: Supplementary file 2 — Supplementary Figure 1. [file 41598_2024_63152_MOESM2_ESM.jpg]

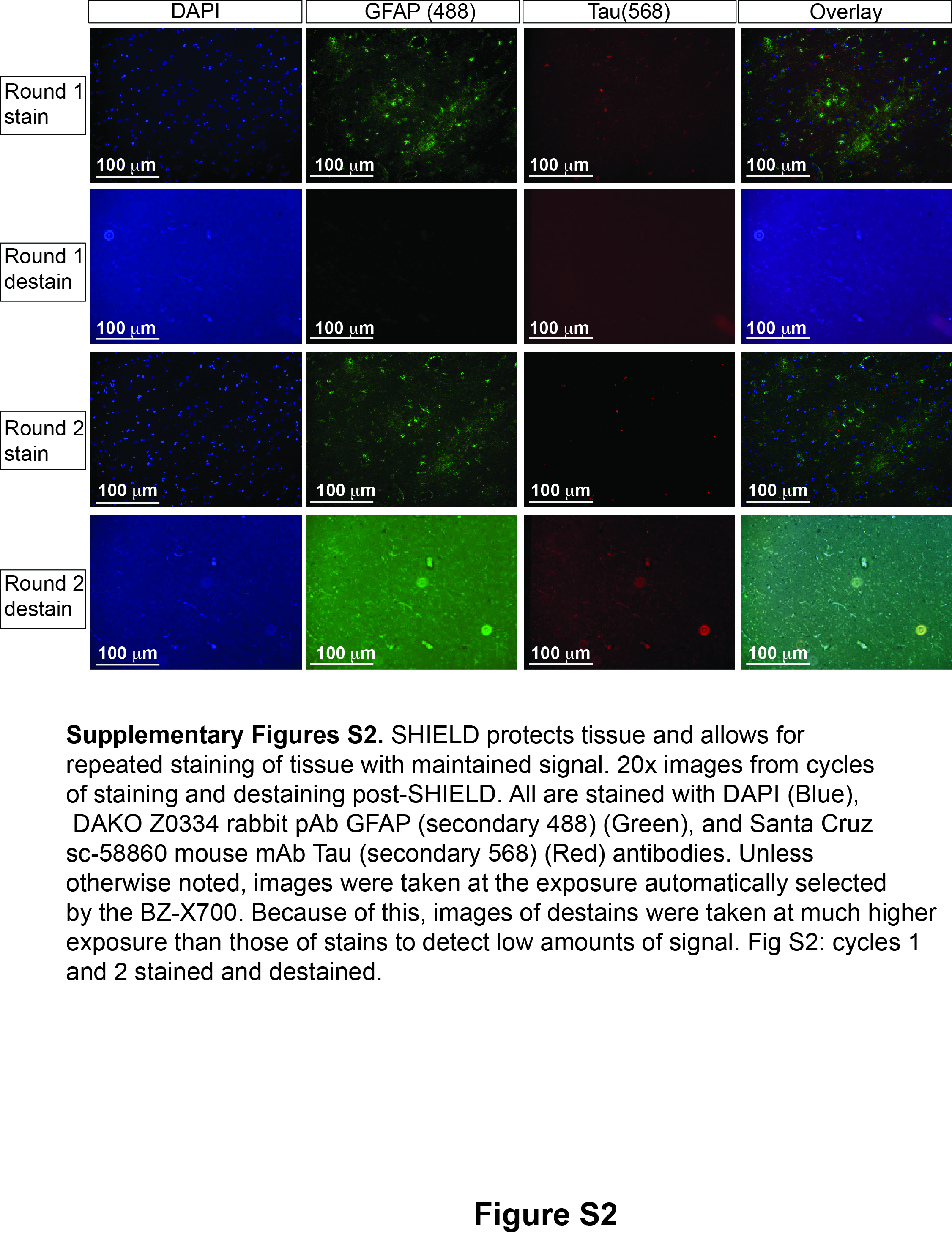

Supplement: Supplementary file 3 — Supplementary Figure 2. [file 41598_2024_63152_MOESM3_ESM.jpg]

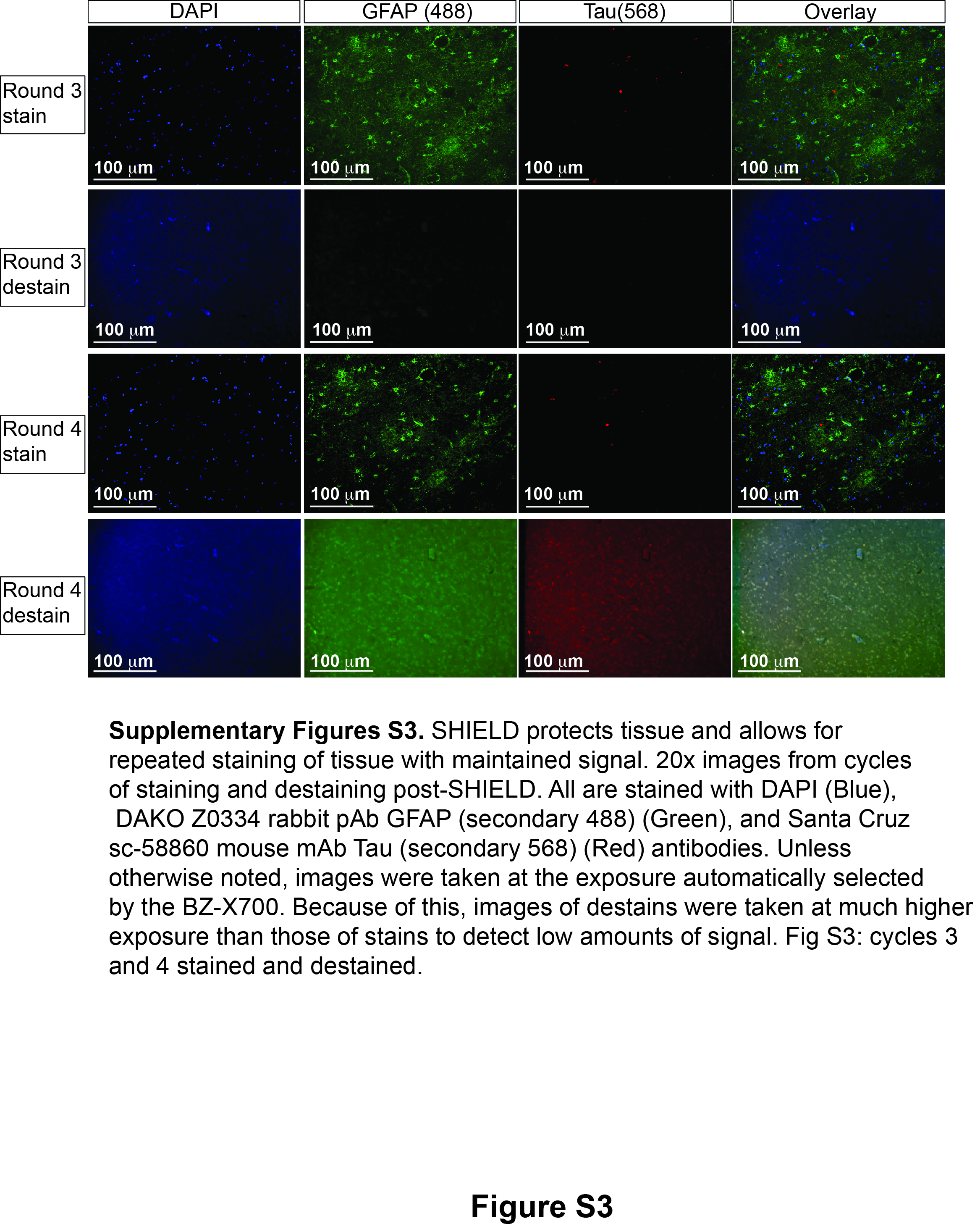

Supplement: Supplementary file 4 — Supplementary Figure 3. [file 41598_2024_63152_MOESM4_ESM.jpg]

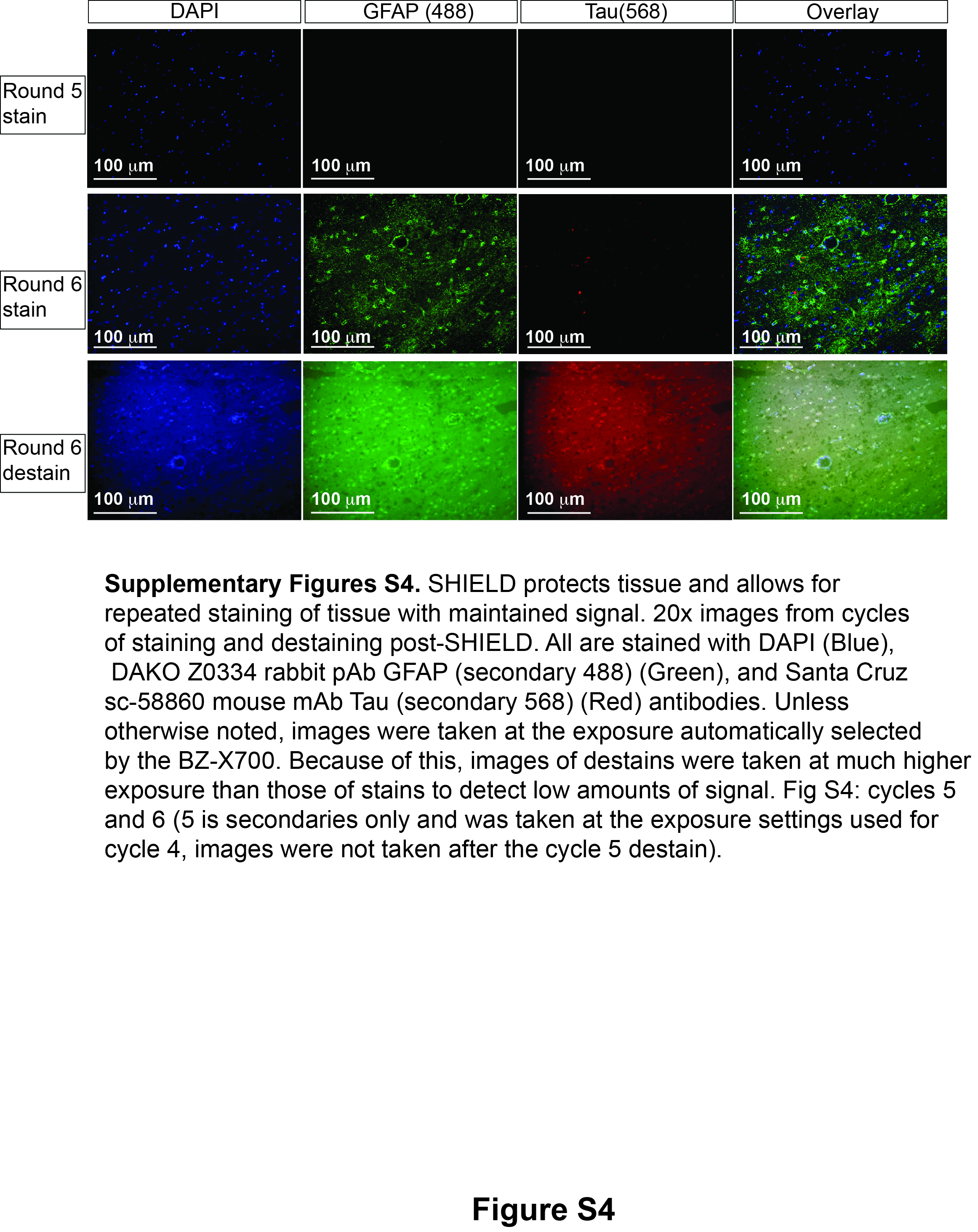

Supplement: Supplementary file 5 — Supplementary Figure 4. [file 41598_2024_63152_MOESM5_ESM.jpg]

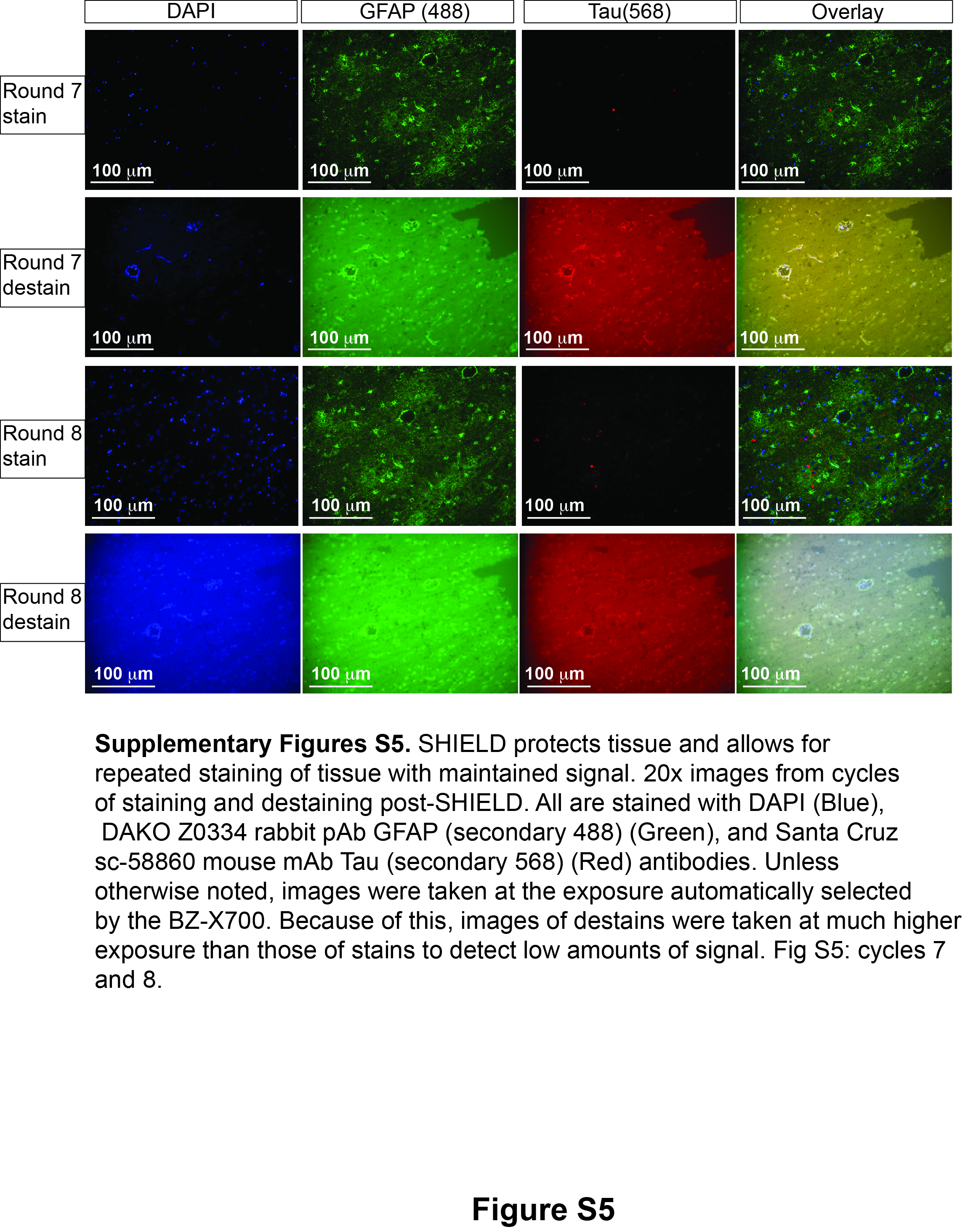

Supplement: Supplementary file 6 — Supplementary Figure 5. [file 41598_2024_63152_MOESM6_ESM.jpg]

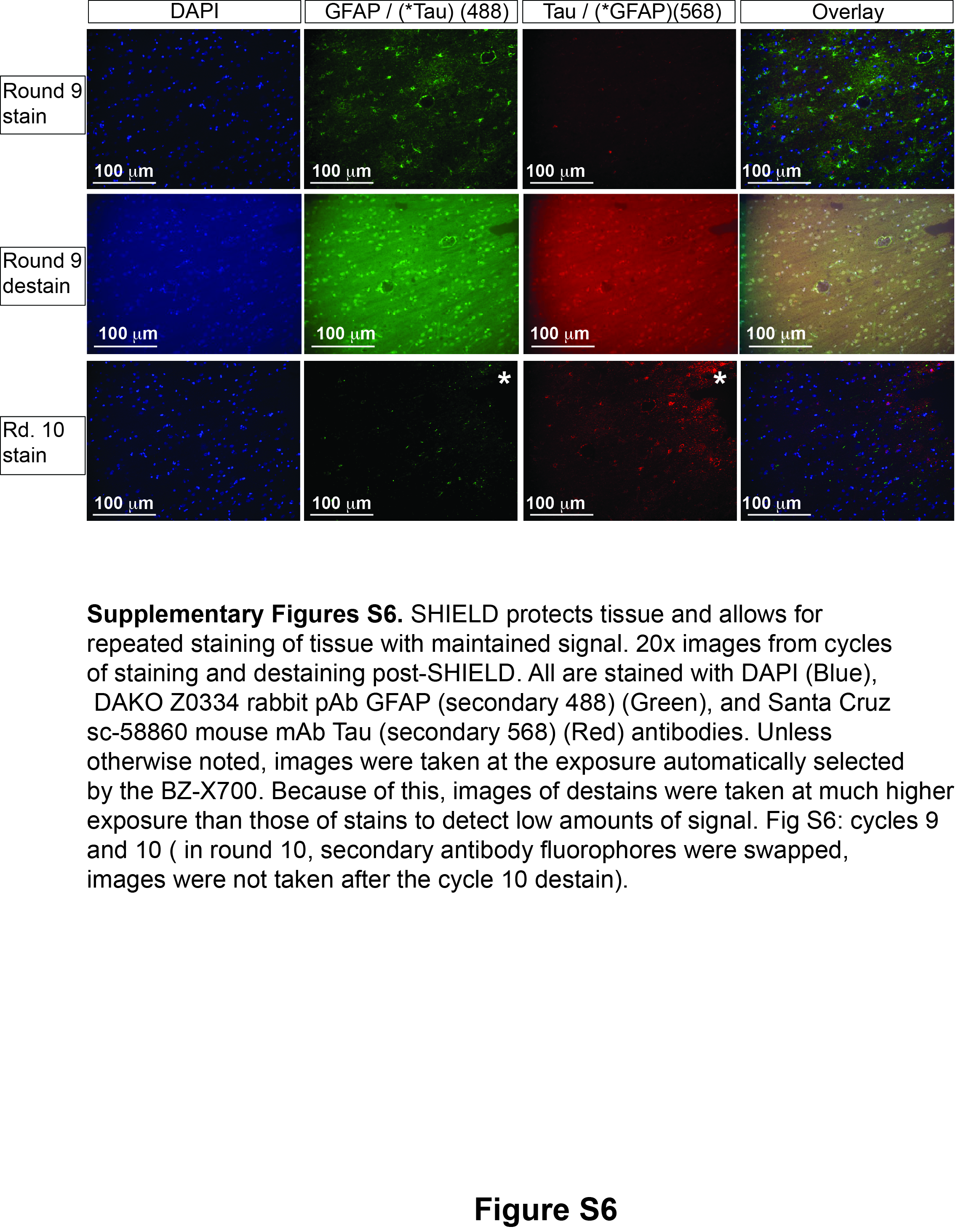

Supplement: Supplementary file 7 — Supplementary Figure 6. [file 41598_2024_63152_MOESM7_ESM.jpg]

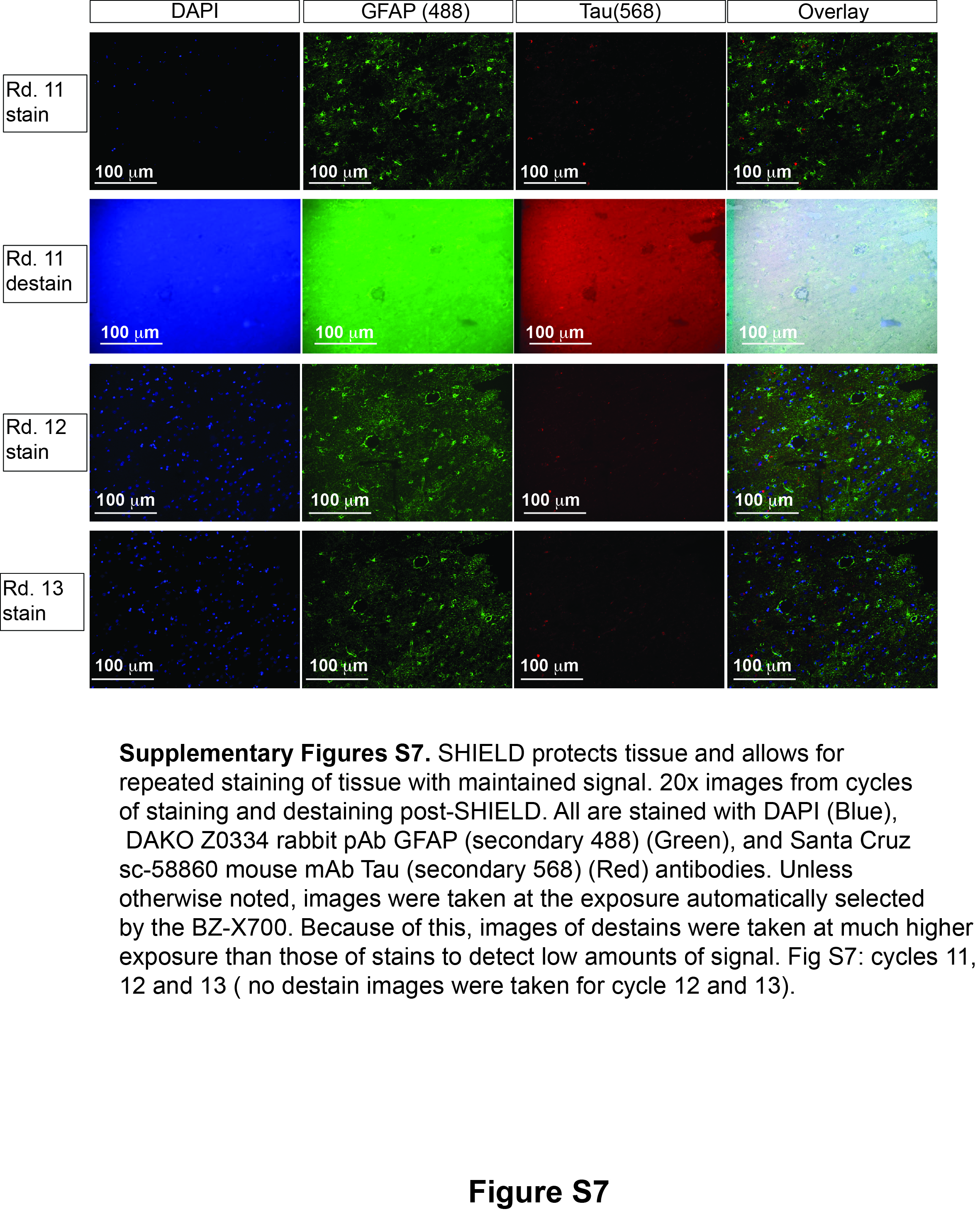

Supplement: Supplementary file 8 — Supplementary Figure 7. [file 41598_2024_63152_MOESM8_ESM.jpg]
